# L-DOPA Is an Endogenous Ligand for OA1

**DOI:** 10.1371/journal.pbio.0060236

**Published:** 2008-09-30

**Authors:** Vanessa M Lopez, Christina L Decatur, W. Daniel Stamer, Ronald M Lynch, Brian S McKay

**Affiliations:** 1 Department of Ophthalmology and Vision Science, The University of Arizona, Tucson, Arizona, United States of America; 2 Department of Physiology, The University of Arizona, Tucson, Arizona, United States of America; 3 Department of Cell Biology and Anatomy, The University of Arizona, Tucson, Arizona, United States of America; Stanford University School of Medicine, United States of America

## Abstract

Albinism is a genetic defect characterized by a loss of pigmentation. The neurosensory retina, which is not pigmented, exhibits pathologic changes secondary to the loss of pigmentation in the retina pigment epithelium (RPE). How the loss of pigmentation in the RPE causes developmental defects in the adjacent neurosensory retina has not been determined, but offers a unique opportunity to investigate the interactions between these two important tissues. One of the genes that causes albinism encodes for an orphan GPCR (OA1) expressed only in pigmented cells, including the RPE. We investigated the function and signaling of OA1 in RPE and transfected cell lines. Our results indicate that OA1 is a selective L-DOPA receptor, with no measurable second messenger activity from two closely related compounds, tyrosine and dopamine. Radiolabeled ligand binding confirmed that OA1 exhibited a single, saturable binding site for L-DOPA. Dopamine competed with L-DOPA for the single OA1 binding site, suggesting it could function as an OA1 antagonist. OA1 response to L-DOPA was defined by several common measures of G-protein coupled receptor (GPCR) activation, including influx of intracellular calcium and recruitment of β-arrestin. Further, inhibition of tyrosinase, the enzyme that makes L-DOPA, resulted in decreased PEDF secretion by RPE. Further, stimulation of OA1 in RPE with L-DOPA resulted in increased PEDF secretion. Taken together, our results illustrate an autocrine loop between OA1 and tyrosinase linked through L-DOPA, and this loop includes the secretion of at least one very potent retinal neurotrophic factor. OA1 is a selective L-DOPA receptor whose downstream effects govern spatial patterning of the developing retina. Our results suggest that the retinal consequences of albinism caused by changes in melanin synthetic machinery may be treated by L-DOPA supplementation.

## Author Summary

Albinism is the loss of pigmentation caused by mutations in one of several different genes that alter pigment synthesis by different mechanisms. In the eye, albinism impairs sensory retina development and causes significant vision problems. Regardless of the genetic mutation that causes albinism, the associated vision problems are the same. Interestingly, none of the pigmentation genes are expressed by the sensory retinal cells affected by albinism but by neighboring, retinal pigment epithelial cells (RPE). Furthermore, loss of pigmentation in RPE somehow leads to imprecise retinal development. To investigate this cellular relationship, we studied OA1, which is encoded by a gene in which mutations cause ocular albinism. OA1 is unique among proteins involved with albinism because OA1 is a potential receptor that could participate in signal transduction rather than being a direct member of the pigment synthesis machinery. We show that the ligand for OA1 is L-DOPA, thus removing OA1 from orphan G-protein coupled receptor (GPCR) status. L-DOPA is a by-product of pigment synthesis, indicating that pigment synthesis and OA1 signaling are intertwined. OA1 signaling is highly selective for L-DOPA, and we show that two closely related molecules, dopamine and tyrosine, bind to OA1 but fail to stimulate signaling. We also show that OA1 signaling controls secretion of a potent neuron survival factor. Taken together, our data suggest that all forms of albinism produce the same retinal defects because of a final common pathway through OA1 signaling with downstream effects on RPE neurotrophic factor secretion.

## Abstract 3

Albinism produces retinal defects, and OA1 is an orphan G-protein-coupled receptor that leads to albinism without acting directly on melanin synthesis. Here the ligand is identified and a mechanism is proposed by which the various forms of albinism signal through OA1, resulting in the same retinal phenotype.

## Introduction

Albinism is a group of inherited genetic diseases in which there is a variable loss of pigmentation in the eye, hair, or skin. When the eye is affected, there are significant alterations in neurosensory retina development that lead to low vision [1–8]. There are two broad classes of albinism, ocular-cutaneous albinism (OCA) and ocular albinism (OA). OCA occurs when all pigmented tissues exhibit hypopigmentation and involves genetic mutations that result in defects in the melanin synthetic machinery [3,7–9]. OA occurs when cutaneous tissues pigment normally, but the ocular tissues are hypopigmented [10,11]. Since the same proteins produce pigment in all tissues, OA most likely results from lack of expression of the melanogenic enzymes in ocular tissue rather than an inability to synthesize melanin, because the other tissues pigment normally.

OA can be linked to at least one gene, *Oa1*, which is found on the X chromosome. *Oa1* encodes a 404–amino acid protein likely to be an orphan G-protein coupled receptor (GPCR), OA1 (GenBank GPR143) [12,13] based upon sequence analysis [14]. Schiaffino et al. have demonstrated that OA1 associates with several G_α_ subunits as well as G_β_, adding further evidence that OA1 is a GPCR [14,15]. Indeed, Innamorati et. al. used a combinatorial expression strategy to illustrate GPCR-like activity from OA1, as well as β-arrestin association, even in the absence of a ligand [16]. This work suggested that OA1 could signal through a Gαq subunit through phospholipase C and inositol triphosphate second messengers. In a yeast-based expression system, Staleva and Orlow have demonstrated GPCR signaling from OA1 that appeared to be activated by a component in the melanosomal compartment [17]. Despite the significant amount of circumstantial evidence that OA1 is a GPCR, confirmation is lacking because no ligand has been identified. Other data have called into question the idea that OA1 is a GPCR. For example, the localization of OA1 as a fully intracellular protein is not typical of GPCRs and suggests that it would be a unique member of the family [14]. OA1 is primarily localized to the endolysomal compartment [14,15,18–21] and melanosomes [11,14,22] rather than the cell surface.

In this study, we investigated the function of OA1 as a potential GPCR. Our hypothesis was that the endosomal localization of OA1 in cultured cells was due to internalization of OA1 in response to an agent in the culture medium. Further, we sought a ligand for OA1 based on the observation that all forms OCA and OA appear to have the same retinal phenotype, indicating that tyrosinase activity and OA1 signaling are coupled upstream of retinal development. Thus, we tested whether tyrosinase activity produces the ligand for OA1. A by-product of melanin synthesis is L-DOPA, which is released to the retina during melanin synthesis in the RPE at a critical time in retinal development [23,24]. Our data suggest that OA1 is a highly selective L-DOPA receptor, and that L-DOPA causes OA1 signaling with the downstream effect of neurotrophic factor secretion by RPE. Thus, we present the first evidence of a ligand for OA1, and provide a mechanism through which either tyrosinase or OA1 deficiency results in changes to retinal development.

## Results

### Cell Surface Localization of OA1

**Figure 1:**
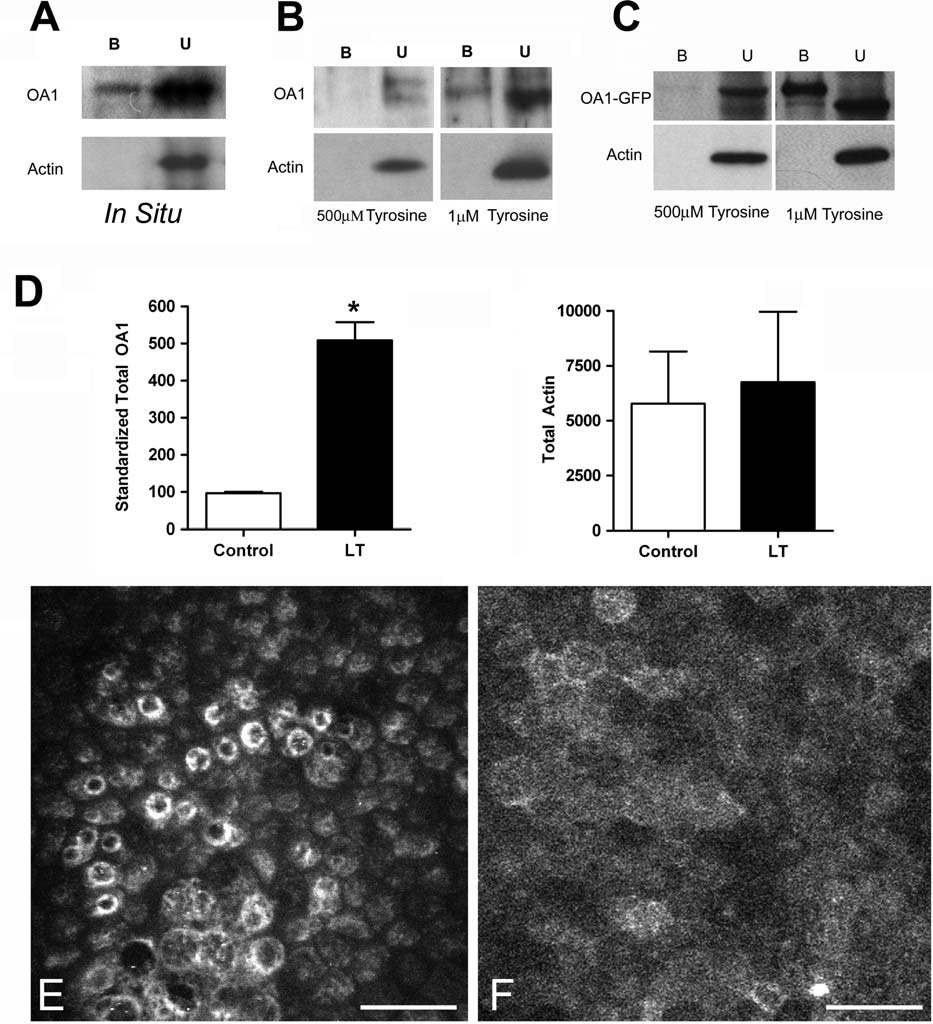
Cell Surface OA1 (A–C) Western blot analysis of proteins bound (B) or unbound (U) to strepavidin-conjugated beads after biotinylation of RPE in situ, cultured RPE (B), or COS cells transfected to express OA1-GFP (C). Blots were probed to visualize OA1 and actin after cell surface biotinylation and fractionation using strepavidin-conjugated beads. For cultured cells (B and C), cells were either maintained in 500 μM (normal DMEM) or 1 μM tyrosine for 3 d prior to analysis. (D) Quantification of western blot analysis by densitometry. OA1 densitometry is shown as the percentage of the control for paired cell cultures, transfected, and then split into two equal groups, one of which was the control, maintained in normal DMEM (control; open bars). The other group was maintained in 1 μM tyrosine DMEM (LT; solid bars) until harvest. Paired *t*-test analysis was used to test whether the difference was significant, and an asterisk (*) denotes *p* < 0.001. Actin, analyzed the same way, showed no differences, and *p* = 0.724. (E and F) Composite confocal microscopy of pigmenting RPE cells maintained in normal DMEM (E) or 1 μM tyrosine (F), and then stained with anti-OA1 antibodies and imaged at 20×. Bar represents 25 μm.

OA1 has previously been localized in pigment granules in situ [22]; however, using transfected cells of various types, OA1 also has been localized to both the plasma membrane [16,17] and the endosomal fraction of cultured cells [14,16–18,20,21]. We began our investigation by determining where OA1 resides in the human tissue, using cell surface biotinylation/western blot strategies. In the human eye, OA1 was present on the apical cell surface of the retinal pigment epithelial cells (RPE) in situ (Figure 1A). Quantification of cell surface, biotinylated OA1 in five human eyes indicated that at least 3.5 ± 0.7% of the total OA1 resided on the apical cell surface of RPE in situ. Access to the biotinylation reagent using eye cup preparations is restricted to the apical surface, so the polarity of OA1 in the epithelium cannot be determined. Further, the total cell surface OA1 is likely underestimated because of the lack of access to the basal cell surface. Blots were also probed with antibodies against actin as a control to verify that cytoplasmic proteins were not biotinylated, and in each experiment, actin was only found in the unbound fraction.

Others have reported that recombinant OA1 and OA1-GFP is almost exclusively localized to the endosomal compartment in cultured cells [14,15,17,18,20–22]. However, when overexpressed [16], or when endocytosis is inhibited [17], OA1 accumulates at the cell surface. Our observation that OA1 protein is present on the apical surface of RPE in situ led us to explore the issue further.

### Effects of Tyrosine on OA1 Expression and Distribution

Endosomal localization of GPCRs occurs normally after exposure to a ligand. Therefore, we investigated whether a ligand for the receptor was present in the standard incubation medium that could drive internalization of OA1. Since the standard culture medium contains 500 μM tyrosine, and tyrosine is the starting material for pigment synthesis, we evaluated the effect of tyrosine on receptor distribution. To test whether tyrosine affected OA1 distribution in cultured cells, we formulated DMEM without tyrosine, and used dialyzed fetal bovine serum. In the presence of tyrosine-free medium, OA1 was detected on the plasma membrane of cultured RPE cells both in the absence (unpublished data), and in medium containing low concentrations of tyrosine (1 μM, Figure 1B). Averaged over five experiments, 4.5 ± 1% of total OA1 protein was observed on the surface of cultured RPE maintained in 1 μM tyrosine, similar to what was observed for RPE in situ. In all experiments, actin was observed in the unbound protein fraction, demonstrating the absence of any cytoplasmic protein in the cell surface assay. Similarly, OA1-GFP expressed in COS cells illustrated a cell surface expression that was tyrosine sensitive (Figure 1C). Quantification of six such experiments indicated significant variability in the amount of OA1 found at the cell surface using transient transfections. The range of OA1 in the bound fraction of transfected cells maintained in 1 μM tyrosine ranged between 5%–40%, unlike the results with the endogenous OA1 protein that were reproducibly approximately 5%.

Not only was the distribution of OA1 in transfected cells sensitive to tyrosine levels in the medium, but total OA1-GFP expression was increased 5-fold in cells maintained in 1 μM tyrosine. To verify that this difference related to OA1 expression rather than cell number, we evaluated actin expression from the paired samples. The data (Figure 1D) presented as optical density units indicate no difference in actin. We also compared the amount of cell surface OA1 between the normal and low-tyrosine groups. Importantly, in the five RPE experiments and six OA1-GFP in COS experiments, we were unable to reproducibly detect OA1 in the plasma membrane fraction of cells in standard medium, similar to that found by others.

The distribution of OA1 in RPE cells also was evaluated by confocal microscopy. OA1 has previously been characterized as an endosomal protein in cultured RPE cells as shown in Figure 1E. In contrast, the distribution of OA1 in low-tyrosine medium was diffuse on the plasma membrane of cultured RPE cells, with little endosomal accumulation (Figure 1F), an observation consistent with the results obtained using biochemical methods.

### L-DOPA as a Natural Agonist for OA1

**Figure 2:**
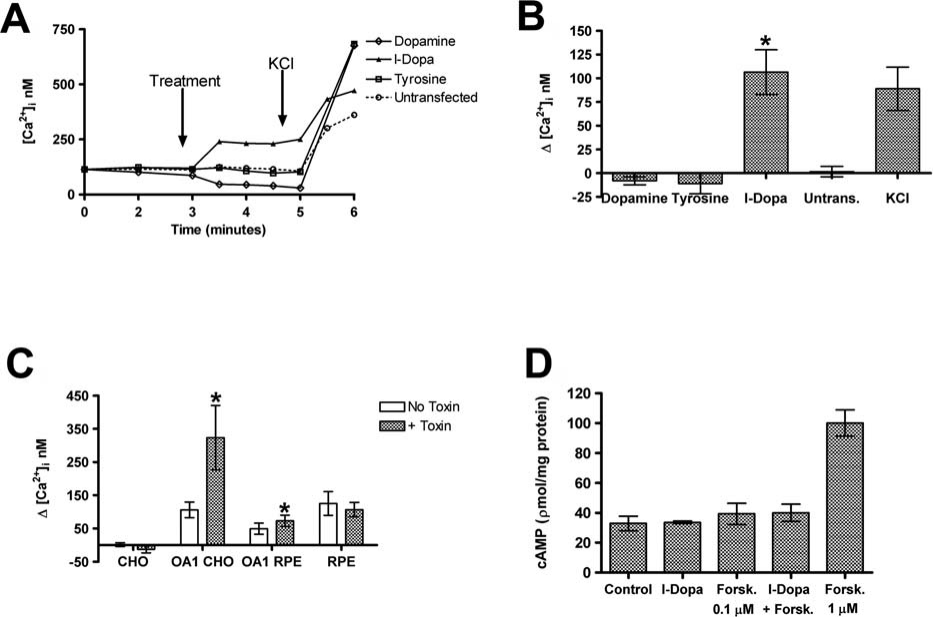
Increased Intracellular Calcium in Response to L-DOPA. (A) Representative traces of [Ca^2+^]i during the time course of the standard experimental protocol in transfected and untransfected CHO cells. After establishment of a stable baseline for 3 min, the test agent was added at 1 μM. At 5 min, KCl was added to serve as a control that the cells were Fura-2 loaded and patent. Identical protocols were performed for both transfected cells and paired untransfected cells. (B) Summary data for [Ca^2+^]i in response to tyrosine, dopamine, and L-DOPA in transfected and untransfected CHO cells. Untransfected cells are shown with L-DOPA treatment. Our experimental control of membrane depolarization with KCl is also shown. Each bar represents data collected from at least ten experiments and is presented as the mean change from baseline [Ca^2+^]i after test agent addition. Error bars represent SD, *t*-test analyses were used to test for significant differences, and an asterisk (*) denotes *p* < 0.01. (C) Analysis of pertussis toxin sensitivity of [Ca^2+^]i increase in cells transfected to express OA1 or RPE that express the natural protein. Data represent mean of at least six experiments for each group of transfected cells and 20 individual experiments for each of the treated and untreated RPE with endogenous OA1 expression. The *t*-test analyses were used to test for significant differences, and an asterisk (*) denotes *p* < 0.01. (D) cAMP was measured in CHO transfected to express OA1. The control group represents transfected, but untreated, CHO cells and the basal level of cAMP in those cells. Cells were treated with 1.0 μM L-DOPA, 0.1 μM forskolin, L-DOPA + 0.1 μM forskolin, and as a positive control, 1 μM forskolin. Results represent the mean cAMP levels observed in at least six experiments in which all experimental groups were analyzed in a paired fashion using replicate monolayers in the same culture plate. Error bars represent the SD of each group, and the only significant difference observed was the increase in cAMP levels after forskolin treatment.

Tyrosinase function in melanogenesis begins with its activity on tyrosine to create L-DOPA, followed by a second reaction to create dopaquinone that leads to pigment formation [25]. Of the intermediates between tyrosine and melanin, L-DOPA has the greatest half-life, and L-DOPA is released into the subretinal space apical to the RPE when melanin synthesis occurs [23,24]. L-DOPA is also the precursor to dopamine, a neurotransmitter produced by dopaneurgic neurons from tyrosine. The release of calcium from intracellular stores is a common downstream effect of GPCR activation by a ligand. Since the expression of OA1 on the cell surface appears to be sensitive to tyrosine, we examined whether tyrosine, or its metabolites L-DOPA and dopamine, could stimulate influx of Ca^2+^ into the cytoplasm in an OA1-dependent manner. CHO cells were transfected with an OA1 expression vector then maintained in DMEM containing 1 μM tyrosine for 48 h, followed by tyrosine-free DMEM for 24 h to facilitate cell surface expression of OA1. Intracellular Ca^2+^ was evaluated using Fura-2, and [Ca^2+^]i was determined by ratiometric imaging [26]. In the absence of any ligand, [Ca^2+^]i was not significantly different between transfected and untransfected cells (Figure 2). Tyrosine and several tyrosine metabolites were tested at 1 μM for an effect on [Ca^2+^]i. As a positive control, we ended each experiment by treatment with 20 mM KCl to depolarize the cell and increase [Ca^2+^]i via activation of voltage-gated channels. This maneuver served to verify the Fura-2 loading and responsiveness of the cells being tested (Figure 2). Only L-DOPA elicited a significant increase in [Ca^2+^]i (Figure 2A). Tyrosine and dopamine had no positive effect on intracellular at [Ca^2+^]i concentrations up to 1 mM (unpublished data). The slight negative effect of 1 μM dopamine was not statistically significant, but it was reproducible among the 11 experiments with dopamine (Figure 2B).

Overexpression of GPCRs in nonnative cell lines can lead to false signal transduction coupling. To verify that OA1 signaling in response to L-DOPA was indeed a natural response, we expressed OA1 in RPE cells (Figure 2C). Results using transfected RPE cells were similar to those achieved with transfected CHO cells. RPE cells transfected to express OA1 responded to 1.0 μM L-DOPA with an increase in [Ca^2+^]i. We next sought to determine whether RPE cells expressing the endogenous OA1 receptor, at endogenous levels exhibited L-DOPA responsiveness. Like all of the transfected cell experiments, RPE expressing OA1 demonstrated an increase in [Ca^2+^]i after treatment with 1.0 μM L-DOPA (Figure 2C).

To further characterize OA1 signaling activity, we used pertussis toxin to distinguish between G_q_ coupled [Ca^2+^]i signaling and G_i_ linked signaling (Figure 2C). In all cells studied, pertussis toxin lowered the basal level of [Ca^2+^]i, indicating its activity on inhibition of the background signaling through G_i_ subunit activity. Pertussis toxin was used in experiments conducted in cells transfected to express OA1 including both CHO and RPE, as well as RPE expressing the endogenous OA1 protein at natural levels. In all transfected cells tested, the measured [Ca^2+^]i response to L-DOPA was greater than in the absence of the toxin (Figure 2), owing largely to the lower initial [Ca^2+^]i. Thus, the signaling through OA1 in response to L-DOPA that results in increase [Ca^2+^]i is not pertussis toxin sensitive and likely G_q_ subunit mediated. We also measured the second messenger cAMP in CHO cells transfected to express OA1 (Figure 2D). Using inactive cells or a submaximal forskolin treatment, the experiments were set up to measure either an increase or decrease in cAMP in response to L-DOPA. In six such experiments, no change in cAMP was observed suggesting neither G_s_ nor G_i_ subunits are involved in OA1 signaling.

**Figure 3:**
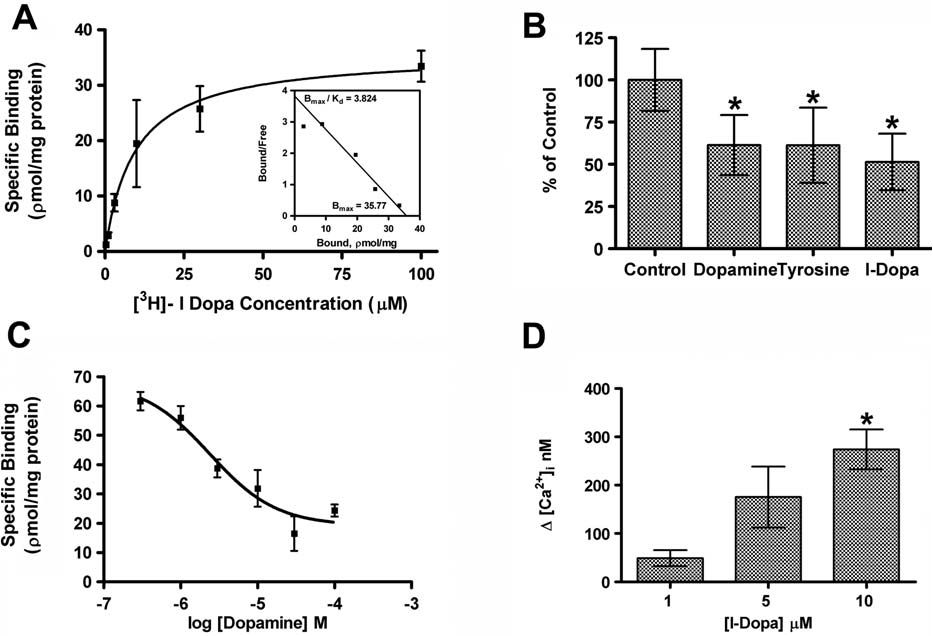
OA1 Ligand Binding (A) Binding kinetics between OA1 and L-DOPA were determined using radiolabeled ligand binding assays. Results represent data collected from five such experiments and are presented as mean specific binding ± SEM. The hyperbolic curve fit exhibited an *r* ^2^ value of 0.994, *K*_d_ was determined to be 9.34 × 10^−6^ M ± 1.14 × 10^−6^ M. The inset Scatchard plot illustrates the kinetics of a single-site binding relationship. (B) We examined the comparative binding of 5 μM [H^3^] L-DOPA to OA1-transfected CHO cells in the presence of 1.0 mM dopamine, tyrosine, or L-DOPA. The data represent mean total binding ± SD for each group. An asterisk (*) denotes *p* < 0.05 when comparing the results between the control group to the binding in the presence of the potential competitive ligands. (C) Competitive interaction between 5 μM [H^3^] L-DOPA and dopamine was assessed to determine whether dopamine functions as an antagonist of OA1 activity. Results indicate that dopamine and L-DOPA compete for the same OA1 binding site, and the data fit the binding model with an *r* ^2^ value of 0.95. The *K*_i_ for dopamine was 2.388 ± 0.266 μM (mean ± SEM), similar to the *K*_d_ for L-DOPA. (D) Dose-dependent OA1 signaling through OA1. Data represent mean increase in [Ca^2+^]i elicited by L-DOPA treatment of the cells at the concentrations given (*n* = 6 for each dose). We used *t*-test analyses to compare between the responses achieved at each dose, and an asterisk (*) denotes *p* < 0.01 for the comparison at 1 and 10 μM.

We utilized standard methods of radiolabeled ligand binding to characterize the interaction between OA1 and L-DOPA (Figure 3A). CHO cells were transfected to express OA1, then binding of L-DOPA was quantified in a concentration-dependent manner, and the results were further characterized by Scatchard plot analysis (Figure 3A, inset). Results illustrate saturable binding of L-DOPA to OA1 expressing cells with a Kd of 9.35 × 10^−6^ M. No specific binding was observed in untransfected CHO cells, indicating that the cells do not have an endogenous L-DOPA receptor (unpublished data). All binding parameters, total, specific, and nonspecific, are shown as supplemental data (Figure S1A). Tyrosine exhibited the potential to interact with OA1, but neither tyrosine nor dopamine stimulated OA1 signaling (see Figure 2). We next used competitive ligand binding to determine whether either tyrosine or dopamine competed with L-DOPA for OA1 binding. At high concentrations (1 mM), both tyrosine and dopamine competed with L-DOPA for OA1 binding (Figure 3B). To further characterize this, we examined the kinetics of the competition between L-DOPA and either dopamine (Figure 3C) or tyrosine (Figure S1B). Dopamine exhibited competitive binding to a single site with L-DOPA with a *K*_i_ of 2.33 × 10^−6^ ± 0.2 × 10^−6^ M. Similar experiments with tyrosine demonstrated inhibition of L-DOPA binding only at high concentrations (Figure S1B). Saturation kinetics were not possible with tyrosine because of its low affinity and insolubility at the high concentrations.

Given the relatively low affinity of OA1 for L-DOPA, we sought to determine whether its signaling activity was dose dependent in the range of this binding affinity. We tested the concentrations in which binding data suggested the steepest rise in association between L-DOPA and OA1, 1.0–10 μM, and results illustrate a concentration-dependent GPCR response as measured by [Ca^2+^]i (Figure 3C). Thus, the activation kinetics of L-DOPA and OA1 matched the concentration range observed in radiolabeled ligand binding experiments.

**Figure 4:**
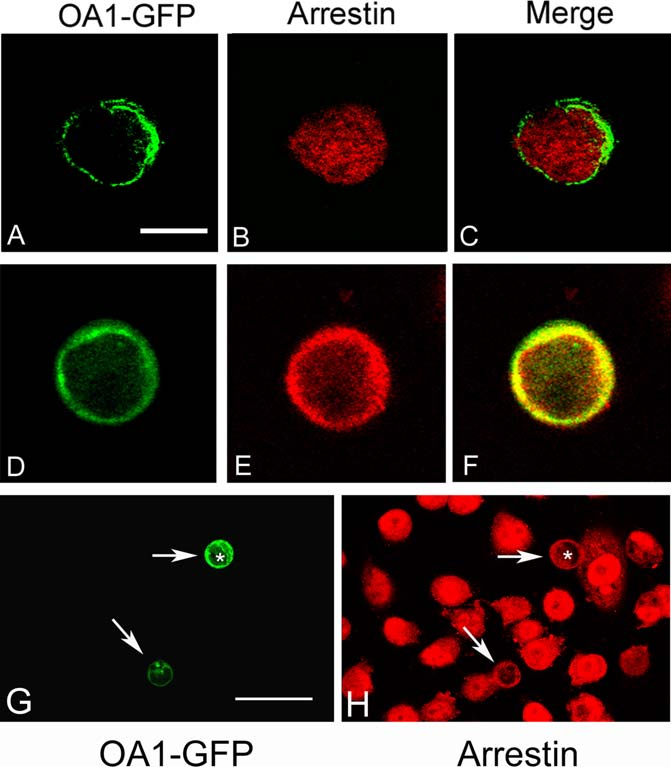
Ligand-Dependent Recruitment of β-arrestin to OA1 (A–F) All images represent 2-μm-thick confocal sections of CHO cells transfected to express OA1-GFP (green). β-arrestin was visualized using immunofluorescence methods (red) prior to addition of L-DOPA (A–C) and after treatment with 1 μM L-DOPA (D–F), and the merged images (C and F) illustrate regions where the two proteins colocalize, at the resolution of white light imaging. Bar represents 10 μm. (G and H) are low magnification of field images of transfected CHO cells, with two transfected cells visible (arrows) (G). The remainder of the cell population is visualized using antibodies to β-arrestin (H) to illustrate that β-arrestin recruitment to the membrane only occurred in the OA1-expressing cells (arrows). Bar represents 25 μm.

In response to ligand binding, GPCRs recruit β-arrestin to the plasma membrane, which is followed by internalization of the ligand–receptor complex [27–33]. We next sought to test the effect of L-DOPA on β-arrestin localization (Figure 4). Cells were transfected to express OA1 and then cultured in 1 μM tyrosine DMEM for 48 h prior to analysis to allow cell surface expression of the protein. Cells were then treated with 1 μM L-DOPA, followed by rapid fixation on ice in cold methanol. Initially, under resting conditions in the absence of an agonist, OA1-GFP was found at the cell surface and β-arrestin was diffuse in the cytoplasm (Figure 4A–4C), with no colocalization between the proteins. After stimulation with L-DOPA, OA1 and β-arrestin were colocalized at the plasma membrane (Figure 4D–4F). Untransfected cells showed no response to L-DOPA treatment (Figure 4G and 4H), illustrating that the L-DOPA effect on β-arrestin distribution was OA1 dependent, similar to results obtained for [Ca^2+^]i.

### Effects of 1-DOPA on PEDF Secretion

**Figure 5:**
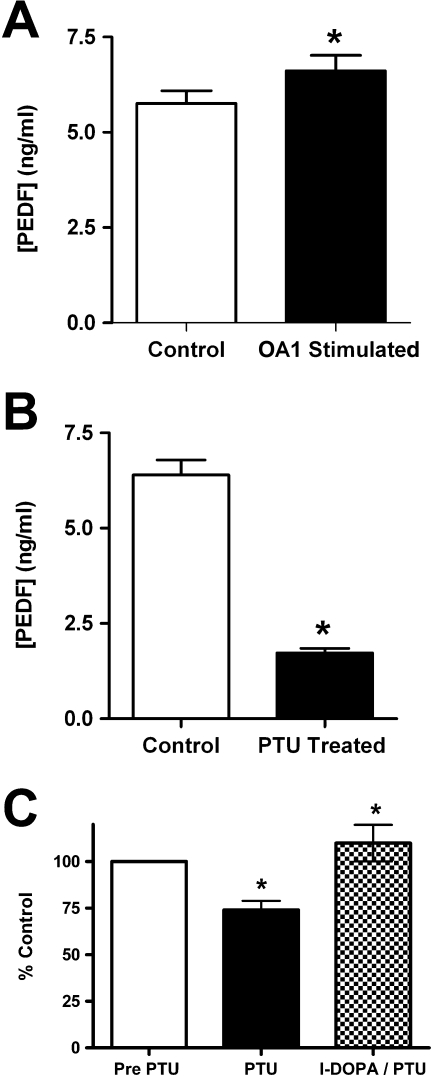
l-DOPA Stimulates PEDF Production in RPE (A) PEDF concentrations were determined by ELISA of cell-conditioned medium. RPE cells were control cells that were without L-DOPA treatment (open bar), or OA1-stimulated cells that were treated with 1 μM L-DOPA prior to being maintained for 3 d in normal DMEM (solid bar). Data are presented as the mean of three experiments conducted in triplicate, error bars represent SD, and an asterisk (*) denotes *p* < 0.01 using a paired *t*-test. (B) PEDF concentrations in conditioned medium from pigmenting RPE determined by ELISA. Cells were either control pigmenting RPE cultures (open bar) or paired cultures treated with phenylthiourea (PTU) at 200 μM (solid bar). Data are presented as the mean of three experiments conducted in triplicate, error bars represent SD, and an asterisk (*) denotes *p* < 0.01 using a paired *t*-test. (C) PEDF concentrations in conditioned medium of pigmented RPE cells treated with PTU and then treated with L-DOPA to stimulate OA1 signaling. ELISA assays were conducted prior to PTU treatment (open bar), then after PTU treatment (solid bar), and then from the same cultures after L-DOPA stimulation (checkered bar). Results are presented as mean ± SD of the value achieved related to that culture of cells. An asterisk (*) denotes *p* < 0.01 when comparing PTU to the control (same culture tested prior to PTU), and L-DOPA/PTU compared to the PTU sample from that same culture.

Mutations in OA1 cause defects in the development of the neurosensory retina. In previous work, we have shown that pigmented RPE secrete significantly more PEDF than nonpigmented RPE [34], and PEDF is a neurotrophic factor with the potential of altering neurosensory retina development [35–41]. Mutations in OA1 cause a loss of pigmentation in the RPE, suggesting that OA1 activity governs RPE pigmentation. Thus, we sought to determine whether L-DOPA stimulation of pigmented RPE cells caused increased secretion of PEDF (Figure 5). This assay is made somewhat more difficult because pigmenting RPE cells produce L-DOPA, which is the agonist for OA1, and OA1 is not readily detectable in nonpigmented cultures of RPE. Thus, we used pigmented RPE to determine whether L-DOPA stimulation increases PEDF expression/secretion. RPE cells were placed in tyrosine-free medium for 24 h and then treated with 1 μM L-DOPA for 1 h. After treatment, the cells were returned to standard medium without exogenous L-DOPA for 3 d. Control cells were not treated with L-DOPA, but the medium was changed at the same time the experimental cells were returned to normal medium. Conditioned medium was collected after 3 d, and PEDF was measured. Results illustrate a significant increase in the secretion of PEDF in pigmented cells treated with L-DOPA when compared to paired, control monolayers of pigmented RPE (Figure 5A). Importantly, this significant increase occurred in cells that were pigmenting and therefore expressed OA1 and had a basal level of PEDF expression.

To determine whether pigmented RPE cells secrete PEDF through an autocrine loop involving tyrosinase activity and OA1 signaling, we used a specific tyrosinase inhibitor phenylthiourea (PTU) to inhibit pigmentation and L-DOPA production (Figure 5B). In these experiments, pigmented RPE cells were either maintained in DMEM, or DMEM containing 200 μM PTU, for 3 d, then PEDF secretion was measured. Pigmented RPE secreted substantial PEDF, but PTU caused a significant decrease in PEDF secretion, indicating that tyrosinase activity is necessary for the high level of PEDF secretion observed in pigmented RPE cells. To verify that it was the lack of L-DOPA in the PTU-treated cells that caused the decreased PEDF secretion, we used three different cultures of pigmented RPE and exposed them to PTU for 48 h, then treated them with 1.0 μM L-DOPA in the continued presence of PTU and measured PEDF after 72 h (Figure 5C). The data are presented as percent of control for this experiment because the cultures used varied in both pigmentation and PEDF expression before the experiment began. PTU-treated RPE responded to the added L-DOPA by increasing PEDF secretion, indicating that the effect of PTU on PEDF secretion is caused be the lack of L-DOPA production when tyrosinase is inhibited.

## Discussion

There is a complex intertissue relationship between the RPE and the neurosensory retina. One aspect of this relationship is centered on RPE pigmentation, and defects in melanin synthesis that result in significant neurosensory retina alterations [8,23,42]. Our data suggest that OA1 and tyrosinase participate in an autocrine loop through L-DOPA that regulates the secretion of at least one potent neurotrophic factor, PEDF. We also suggest that the pathologic changes in retinal development that occur in albinism may result from changes in the activity of the OA1 signaling pathway. Reduced OA1 signaling activity can be caused either directly through OA1 mutations or indirectly through changes in L-DOPA production by tyrosinase activity. Thus, we hypothesize that the similar retinal phenotypes that accompany the diverse forms of albinism can be reconciled to a single common pathway, OA1 signaling.

In our study, we observed OA1 on the apical surface of human RPE in situ. Previous reports have suggested that OA1 in mice is localized to the melanosome [22], and in cultured cells to the endosomal compartment [15–18,20–22,43]. Our results from in situ RPE preparations indicate that OA1 is distributed to the apical surface of the RPE. The limited quantities of OA1 on the surface of the RPE (∼3.5% of total OA1) may account for the lack of observation of the protein in previous studies in which immunogold electron microscopy was used. Like many cell surface GPCRs, OA1 is not an abundant protein.

The endosomal localization of OA1 reported in previous studies using cultured cells was reproduced in this study for both the endogenous protein and the transgenic protein. When it was tested in normal culture medium, we found little detectable OA1 protein on the cell surface, in agreement with all previous work. However, reduction of tyrosine in the medium caused a modest increase in cell surface receptor accumulation of both the endogenous and recombinant OA1 proteins. This suggests that the distribution of OA1 to the cell surface in cultured cells is sensitive to tyrosine. A previous study has demonstrated OA1 could be localized to the cell surface when endocytosis is inhibited [17], and we observed OA1 on the apical surface of human RPE in situ. We suggest OA1 is a cell surface GPCR, but is a target for endocytosis that may be stimulated by tyrosine or tyrosine metabolites. In this regard, our results differ from past reports of OA1 localization that have classified OA1 as a unique type of intracellular GPCR. Most GPCRs are cell surface proteins that are internalized by a variety of signals, and our data suggest OA1 is similar to most other GPCRs.

OA1 signaling activity was stimulated by L-DOPA, but not by either its precursor, tyrosine, or its neuronal metabolite dopamine. This result suggests an exquisitely sensitive receptor activity able to distinguish between closely related molecules; after all, L-DOPA and tyrosine differ by a sole hydroxyl group. OA1 is sensitive to tyrosine, as tyrosine causes an intracellular localization of OA1 in cultured cells. However, we noted no signaling response to tyrosine, and competition binding studies suggest that tyrosine has a low affinity for OA1. Our data suggest that the continuous exposure of cells to high concentrations of tyrosine present in normal medium is sufficient to result in internalization of OA1, but it is unlikely to result in measurable OA1 activation. We found strong evidence of a single-site competitive interaction between L-DOPA and dopamine. The *K*_i_ observed for dopamine was similar to the *K*_d_ observed for L-DOPA, suggesting that the affinity for the two tyrosine metabolites is similar. Our results illustrated a slight, but reproducible, decrease in OA1 signaling from dopamine, suggesting that dopamine may be an effective antagonist or inverse agonist for OA1.

As an orphan GPCR, its signaling pathway has not previously been identified. In this study, we illustrate that OA1 signaling in response to L-DOPA causes an increase in [Ca^2+^]i. Our data illustrate that the increased [Ca^2+^]i observed in response to L-DOPA was insensitive to pertussis toxin, and we measured no effects on cAMP, indicating that OA1 is likely signaling through a G_q_ subunit. Previous work has suggested that OA1 can associate with multiple subunits in transfected cells, including members of the G_o_, G_i_, and G_q_ subunit families. Innamorati et al. has shown that spontaneous activity of overexpressed OA1 is likely signaled through a Gq subunit [16]. Our data indicate that ligand-dependent signaling from endogenous OA1 in RPE most likely occurs through a G_q_-mediated pathway, and we observed no promiscuous coupling activities when comparing OA1 overexpression in CHO and RPE to natural OA1 expressed in RPE. Interestingly, two overactive mutant forms of Gq subunits cause hyperpigmentation in skin and hair [44], but whether they have an effect in RPE is unknown. RPE and cutaneous melanocytes use the same enzymes to produce pigmentation but differ in their control of melanogenesis. A recent report suggests that OA1 may signal through Gαi3, because the retinal phenotype of OA1^−/−^ and Gαi3^−/−^ are similar [45]. That study provided no data regarding interaction or signaling between Gαi3 and OA1, and our results do not support OA1 signaling through Gαi3. However, both OA1 and Gαi3 could have activity in convergent pathways that govern some part of the complex system of retinal development.

The response of OA1 to L-DOPA was measured in three ways, increased [Ca^2+^]i, recruitment of β-arrestin to plasma membrane OA1, and the increased secretion of PEDF. In addition, we have inhibited the activity of tyrosinase in pigmented RPE, which inhibits L-DOPA production and observed a decreased secretion of PEDF. Taken together, these studies present a strong argument for a productive ligand:receptor relationship between L-DOPA and OA1. Further, our data suggest selectivity among tyrosine and its metabolites, with only L-DOPA being a productive ligand for OA1. We have determined the binding kinetics between OA1 and L-DOPA, and observed a typical one-site receptor:ligand relationship between the two. The binding affinity between OA1 and L-DOPA, with a *K*_d_ in the micromolar range, is not uncommon for an endogenous ligand:receptor relationship. Future identification of a specific, high-affinity antagonist for OA1 will aid in further biochemical characterization of the interaction between OA1 and L-DOPA, and be useful in determining whether dopamine is an inverse agonist.

We have illustrated the selective activation of OA1, an orphan GPCR, by L-DOPA, an intermediate product of melanin synthesis. We have also illustrated that OA1 activity stimulates PEDF secretion by RPE, a molecule that has the potential to support normal retinal development [40,41]. In humans, this suggests that pharmacologic intervention through OA1 activation could be useful for albinism caused by defects in the melanogenic machinery (OCA 1–4). Unfortunately, our data also suggest that OA1 is necessary for such pharmacologic intervention, and mutations in *Oa1* are the most common cause of albinism.

## Materials and Methods

### Cell culture.

RPE were isolated as described [46] and maintained in Dulbecco's modified essential medium (DMEM) supplemented with 5% fetal bovine serum (FBS). For experiments in which tyrosine concentrations were lowered, we used custom manufactured DMEM produced without tyrosine by JRH Biosciences. Dialyzed FBS was purchased from Invitrogen.

COS-7 and CHO cells were obtained from ATCC and cultured in DMEM supplemented with 5% FBS. For analysis of OA1 distribution, cells were cultured in tyrosine-free DMEM supplemented with 1 μM tyrosine, 5% dialyzed FBS for 2–4 d, then tyrosine-free medium as described for the experiment.

### Cell surface biotinylation.

*Human RPE in situ.* Human eyecups were produced by dissection approximately 2 mm anterior to the equator and removal of the anterior segment. The vitreous and retina were removed without impairing the underlying RPE monolayer, and the retina was cut at the optic nerve head. The resulting eyecups with RPE exposed were rinsed three times with reaction buffer (100 mM NaCl, 50 mM NaHCO_3_ [pH 8.0]) and then filled with Sulfo-NHS-LC-Biotin (1 mg/ml) two times for 30 min. The reaction was stopped with TG buffer (25 mM Tris, 192 mM glycine [pH 8.3]) and then the cells were harvested in lysis buffer (2 mM EDTA, 1% Triton X, and 1% Tween 20 in Tris Base Saline Buffer) containing Halt Protease Inhibitor Cocktail. Intact cells and pigment granules were removed by centrifugation at 14,000 rpm for 20 min. Biotinylated proteins were captured overnight with immobilized streptavidin beads and then mixed with 4× reducing buffer (250 mM Tris [pH 6.8], 8% SDS, 40% glycerol, 20% beta-mercaptoethanol, 0.08% bromophenol blue). The OA1 protein was separated on a 10% SDS-PAGE gel and identified by a using a polyclonal rabbit OA1 antibody for western blot analysis. Paired western blots were probed with a monoclonal antibody directed against actin.

Cultured cells. RPE and transfected cells were maintained in DMEM containing tyrosine concentrations described for the experiment. Cultures were rinsed three times in reaction buffer, then biotinylated as described above for the in situ preparation.

### Cloning of *Oa1*.

A cDNA library was constructed from pooled tissue from six human donor eyes. Total RNA was harvested using Trizol reagent, then cDNA was synthesized using Poly-T primers for the first-strand synthesis, and random hexamers for the second strand. Following cDNA synthesis, RNA was removed using RNase A. The coding sequence for OA1 was obtained by PCR using terminal primers that added restriction sites to the 5′ and 3′ ends and removed the native stop codon. The PCR product was ligated in frame with GFP in the pEGFP N-1 vector (Clontech). The sequence was verified by automated sequencing in both directions over the entire sequence.

### Immunocytochemistry.

Cells on slides were fixed with 3% paraformaldehyde at room temperature, rinsed with 0.1% Triton X-100 in 10% milk in TBST, and then blocked with 10% milk in TBST. β-arrestin was visualized using a polyclonal antibody directed against β-arrestin, and incubated overnight at 4 °C. Cover slips were mounted using 50% glycerol, and immunostaining was analyzed by optical sectioning using a Nikon Eclipse E800 laser scanning confocal microscope powered by Compix Confocal Imaging Systems software (Simple PCI Version 4.0.6.1605). Three-dimensional analysis of OA1-GFP and β-arrestin distribution was performed in ImageJ 1.32.

### Measurement of [Ca^2+^]i.

OA1-GFP expressing CHO cells plated on glass cover slips were rinsed in Ca^2+^-containing HEPES-buffered Hanks Balanced Salt Solution (HBSS) (pH 7.45) and then incubated with 2.5 μM Fura-2 (solubilized in anhydrous dimethylsulfoxide and 0.002% pluronic acid) for 20 min at 37 °C, 5% CO_2_.The Fura-2–loaded cells were rinsed with HBSS for 15 min at 37 °C, 5% CO_2_ to allow for full cleavage of the dye to its active form. Each cover slip was incubated in 1 ml of HBSS in a chamber held at 37 °C on the stage of an inverted Olympus IX70 microscope equipped with a 40 × 1.35 NA UV-fluor objective.

Using a filter wheel, excitation light from a 200 W Xe bulb was passed alternately through 340- and 380-nm filters. A 10-nm bandpass filter, centered at 510 nm, selected for the emitted fluorescence that was passed to a CCD camera (Photometrics CH-250).

For each experiment, image pairs were taken every minute for the first 3 min, which established a stable baseline. L-DOPA (1 μM final concentration) was then added, and image sets were taken every 30 s for the next 3 min. Finally, KCl (20 mM final concentration) was added 1 min before completion of each experiment as a positive control to establish that the cells were loaded with Fura-2. The same was repeated independently for tyrosine and dopamine (both at 1 μM final concentration).

Using a Silicon Graphics Personal IRIS computer, the 340/380-nm ratio was computed for each pixel within a cell, and then analyzed using Microsoft Excel version 4.0 (Microsoft). Once the 340/380-nm ratio was determined, each ratio was normalized to 1 (ratio at time zero divided by itself), and then the free ion concentration was calculated using the following equation:


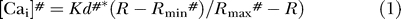


in which *R*, *R*_min_, and *R*_max_ are the measured, minimum, and maximum ratios, respectively. *R*_max_ represents the ratio of fluorescence intensity of ion-sensitive wavelengths under fully deprotonated conditions, whereas *R*_min_ is the ratio for the dye when it is fully protonated. In the case of Fura-2, *R* increases with increasing Ca^2+^; hence *R*_min_ represents Fura-2 in the absence of Ca^2+^ (Ca^2+^ < 1 nM), whereas *R*_max_ represents the Ca^2+^-Fura-2 chelate as previously described [26]. *R*_min_, *R*_max_, and *K*_d_ were determined in independent experiments in Fura-2–loaded cells, and subsequently utilized for calculation of free Ca^2+^ for the experimental procedures.

### Radiolabeled ligand binding.

CHO cells that were transfected to express OA1-GFP were plated into 24-well plates. Cells were chilled to −2 °C, then rinsed in cold binding buffer, 25 mM Tris, 150 mM NaCl, 5 mM EDTA, 5 μM digitonin (pH 7.45). Cells were incubated for 2 h in binding buffer containing [^3^H]-l-DOPA (Moravek Biochemicals) at concentrations between 10^−4^ M to 10^−9^ M. The temperature was not allowed to exceed −2 °C at any step of the assay. Controls included assays conducted on nontransfected CHO, and specific binding was determined by competition with excess unlabelled L-DOPA at 10^−3^ M. Bound L-DOPA was quantified by scintillation spectroscopy.

### Measurement of cAMP.

Cells were pretreated with forskolin (15 min) and then challenged with L-DOPA using an assay setup as previously described [47]. After 1 min of ligand exposure, cells were scraped into ice-cold buffer, boiled, and then centrifuged. Equivalent volumes, 50 μl, of supernate and ^3^H-cAMP (New England Nuclear) were then combined with 100 μl of cold PKA. After 2 h, the solution was passed over activated charcoal, and supernates were counted in a scintillation counter. Results were compared to those achieved using a standard curve, instead of cytosol, produced using 50 μl of cAMP 0.25–32.0 pmol/50 μl.

## Supporting Information

Figure S1 Supplemental Data (A) Data represent mean ± standard error of the mean (SEM) of bound [3H]-l-DOPA in all fractions, total, specific, and nonspecific. Nonspecific binding was determined by measuring radiolabeled-l-DOPA bound in the presence of excess unlabeled L-DOPA (1 mM). Specific binding at each given concentration is determined by subtracting the measured nonspecific binding from the measured total binding.
(B) The figure illustrates competitive interaction between tyrosine and L-DOPA, measured using increasing concentrations of tyrosine and 5 μM [H^3^] L-DOPA. Each data point represents the mean data from five replicate wells, and the error bars represent standard deviation (SD). Data illustrate that tyrosine competes for binding with L-DOPA, but with a low affinity. Our results suggest tyrosine has a *K*_i_ of 52.9 μM, and fits the single-site binding model with an *r* ^2^ value of 0.85. Saturation could not be achieved because of the limited solubility of tyrosine.
(682 KB TIF)

## Abbreviations

GPCR: G-protein coupled receptor
OA: ocular albinism
PTU: phenylthiourea
RPE: retinal pigment epithelial cells
