# Supplementary figures and images for: L-DOPA Is an Endogenous Ligand for OA1
Source: PLoS Biol. 2008 Sep 30;6(9):e236. doi: 10.1371/journal.pbio.0060236 (PMC2553842; doi:10.1371/journal.pbio.0060236)

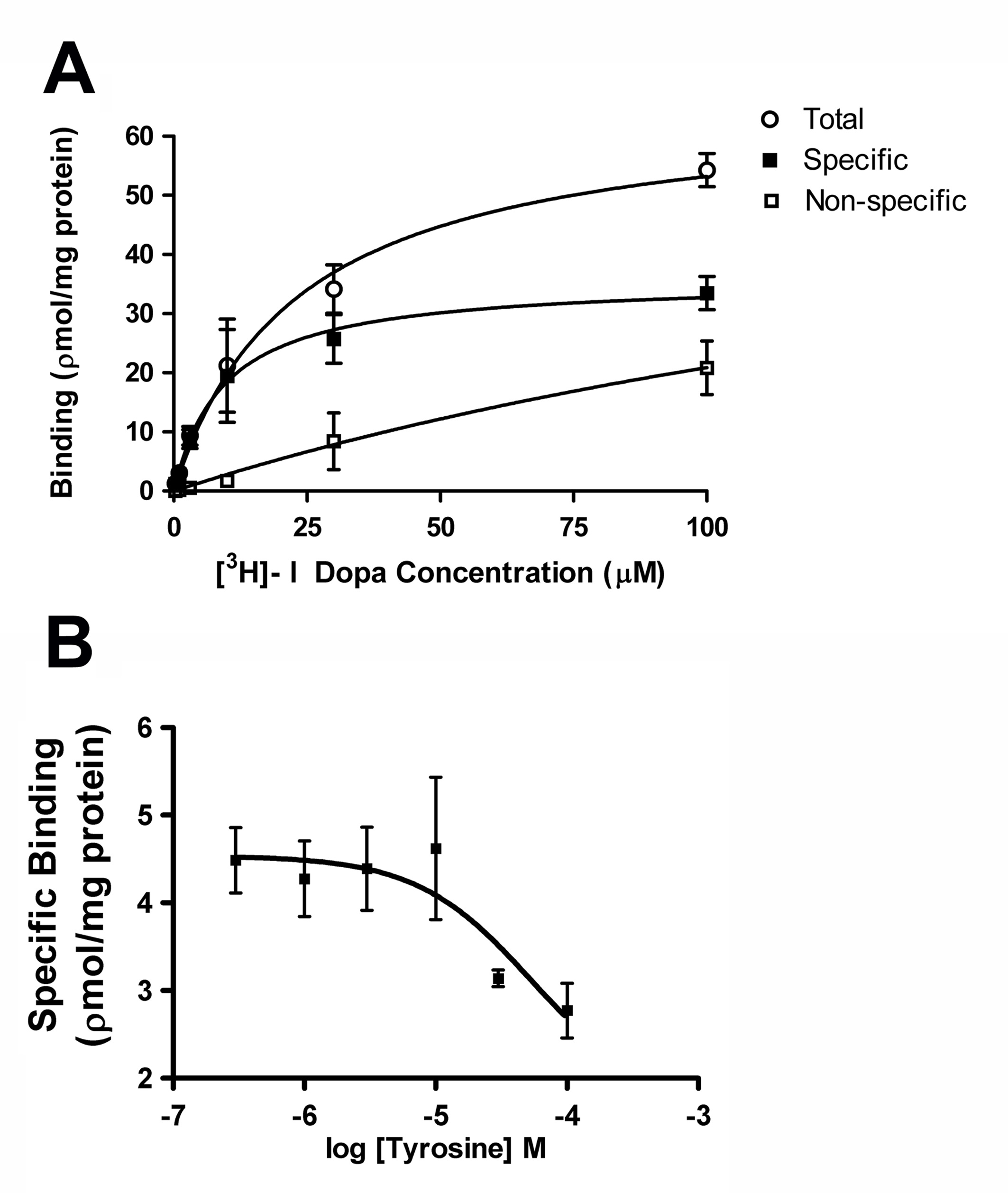

Supplement: Figure S1 — (A) Data represent mean ± standard error of the mean (SEM) of bound [3H]-l-DOPA in all fractions, total, specific, and nonspecific. Nonspecific binding was determined by measuring radiolabeled-l-DOPA bound in the presence of excess unlabeled L-DOPA (1 mM). Specific binding at each given concentration is determined by subtracting the measured nonspecific binding from the measured total binding. (B) The figure illustrates competitive interaction between tyrosine and L-DOPA, measured using increasing concentrations of tyrosine and 5 μM [H3] L-DOPA. Each data point represents the mean data from five replicate wells, and the error bars represent standard deviation (SD). Data illustrate that tyrosine competes for binding with L-DOPA, but with a low affinity. Our results suggest tyrosine has a K i of 52.9 μM, and fits the single-site binding model with an r 2 value of 0.85. Saturation could not be achieved because of the limited solubility of tyrosine. (682 KB TIF) [file pbio.0060236.sg001.tif]
